# Environmental factors affecting the BMI of older adults in the Philippines spatially assessed using machine learning

**DOI:** 10.1016/j.heliyon.2024.e40904

**Published:** 2024-12-06

**Authors:** D.K. Mendoza, A.B. Araza, L.D. Groot, M. Mensink, R.C. Tan

**Affiliations:** a Department of Science and Technology - Food and Nutrition Research Institute, Taguig, Metro Manila, Philippines; b Earth Systems and Global Change, Wageningen University and Research, Wageningen, the Netherlands; c IMPACT R&D, 47 Razburg, San Agustin Bay, Laguna, Philippines; d Division of Human Nutrition and Health, Wageningen University and Research, Wageningen, the Netherlands

**Keywords:** Body mass index, Obesity, Malnutrition, Machine learning, Spatial data, Older persons, Philippines

## Abstract

This study aimed to assess the environmental variables affecting the Body Mass Index of older adults at neighborhood levels (1 ha) while mapping probability distributions of normal, overweight-obese, and underweight older adults. We applied a data-driven method that integrates open-access remote sensing products and geospatial data, along with the first nutritional survey in the Philippines with geo-locations conducted in 2021. We used ensemble machine learning of different presence-only and presence-absence models, all subjected to hyperparameter tuning and variable decorrelation. The cross-validated ensemble model was found to have AUC=0.76-0.93 and TSS =0.45-0.81, which indicates that the models are performing better than random chance. We found that neighborhoods with (a) short distances to the main city, (b) short distances to roads, and (c) with densest road network all drive overweight-obese cases. The latter (c) contrasts the findings in Western developed countries because of delimiting factors in a tropical developing country related to active public transport, crime, weather, the walkability of roads, and even the COVID-19 restrictions during the time of the surveys. The probability distribution maps revealed that the older adults in the Philippine case cities were mostly overweight-obese, especially within and nearby city centers. We finally showed priority neighborhoods for intervention and local policy implementation, providing valuable insights for local government units.

## Highlights

• Malnutrition maps of Filipino older adults (>60 y.o.) derived using spatial ensemble machine learning.
• Short distances to roads and city centers, and dense road networks drive overweight-obesity.
• Hotspots of overweight-obese mapped and identified for targeted interventions.
• Use of the first comprehensive nutritional surveys in the country with geo-locations in three case cities.

## 1 Introduction

The global population of older adults (>60 years old) has been increasing faster than any other age group in most parts of the world [1]. Various health risks increase with age, including malnutrition, a condition when an individual is below or above the normal body mass index due to a number of physical, psychological, social and environmental risk factors [2]. Like many countries, the Philippines is experiencing an increasing number of older adults and malnutrition [3] under limited healthcare resources [4].

Environmental risk factors have been associated with malnutrition. Duante et al. (2019), based on the 2013 Philippine National Nutrition Survey, reported a higher prevalence of overweight and obesity among older adults residing in urban areas compared to those in rural areas [5]. Similarly, Lopez et al. 2016 found an association between adult obesity in highly urbanized cities of the Philippines and the built environment factors such as the number of local commercial establishments and health centers [6]. Dahly and colleagues spatially clustered overweight and obese men in the Philippines, and such clustering allowed the identification of obesity hotspots and their relationship with urbanization [7]. Using provincial surveys, Salvacion in 2017 found that malnutrition was driven by socioeconomic, topographic, and climatic factors in one of the Philippine province islands [8]. While these studies analyzed malnutrition data at aggregated levels e.g., provinces, examining the associations between environmental risk factors and malnutrition at the neighborhood level could provide clearer insights and enable more targeted interventions [9].

The availability of data for both environmental factors and malnutrition is rapidly increasing [10]. Remote sensing data of environmental variables at high spatial resolutions are continuously increasing, such as gridded population data and vegetation greenness indices. Similarly, Geographic Information System (GIS) data like OpenStreetMap, which provides up-to-date data on the built environment such as roads, buildings, and amenities, are publicly available. Malnutrition data is increasingly available through national surveys and other sources such as non-governmental and humanitarian censuses. Recent malnutrition data can more readily incorporate spatial properties by geo-locating surveyed individuals or households using GPS devices or smartphones, enabling spatial analysis and interpretation. The use of geo-located malnutrition surveys and high-resolution environmental data enables the mapping and analysis of malnutrition among older adults at neighborhood scales. Nguyen et al. 2017 examined neighborhood characteristics and showed more physically active had lower obesity prevalence [11]. While mapping neighborhood-level malnutrition and environmental data is promising, there could be precautions in implementing such a data-driven approach. Environmental variables often have non-linear associations with malnutrition particularly obesity [12]. Traditional models like linear or logistic regression may not capture these relationships well, as they are designed for linear associations and often struggle with high-dimensional data.

Machine learning (ML) methods not only capture complex and non-linear relationships between malnutrition and the environment but also allow malnutrition mapping at the scale of map spatial resolution i.e., environmental variables [13], [14]. This can allow producing a map of the probability distribution (occurrence) of older adults who are underweight, overweight, or obese. ML models also allow for the identification of certain environmental data that are most associated with malnutrition. Diou et al. (2019) [15] discussed how ML-based malnutrition mapping and its association with drivers can advance research opportunities in malnutrition. Currently, there are limited ML-based malnutrition mapping studies and current studies were all piloted in developed western cities. For instance, Sun et al. (2020) [16] applied ML to map malnutrition among older adults in New York City, incorporating socioeconomic and environmental factors. Such a study in developing countries such as the Philippines, is needed to target and prioritize those who need interventions at neighborhood levels of cities.

ML methods for high-resolution malnutrition mapping are still challenged by limited geo-located surveys and how they are sampled. Once small, incomplete, and unrepresentative samples are used for fitting ML models, the models tend to overfit and produce misleading results [17]. Aside from these sampling issues, the choice of the ML model considering >20 possible options is not straightforward. While the common models are based on neural networks and decision trees such as random forest and gradient boosting (ML models), a fit-for-purpose but never been used ML for malnutrition mapping is maximum entropy (MaxEnt). MaxEnt is a commonly used ML species distribution model that can be reliable even when using small sample sizes [18]. The recent inter-comparison study of Valavi et al. 2022 [19] revealed top-performing models to be MaxEnt, tuned ML models (best model parameter combination after simulations), and an ensemble of ML models. An ensemble model typically outperforms individual models in terms of mapping potential distributions [20]. This includes ensembles of both presence and presence-absence models [21], [22].

Using an ensemble of different ML models, we aimed to produce neighborhood-level maps (1 ha) of underweight, overweight-obese, and even normal-weight older adults in three case cities in the Philippines while assessing their associations with different environmental risk factors. This was enabled by the first comprehensive nutritional surveys in the country with geo-locations, allowing associations with open-source remote sensing and geospatial data for detailed spatial characterization. We specifically aim to:

1. Develop an ensemble of different ML models of older adults' malnutrition using geo-located household surveys and spatial environmental data;
2. Associate the environmental risk factors to malnutrition and assess the consistency of results among cities; and
3. Map the probability of occurrence and hotspots of malnutrition among older adults.

## 2 Materials and methods

### 2.1 Study areas

**Figure 1:**
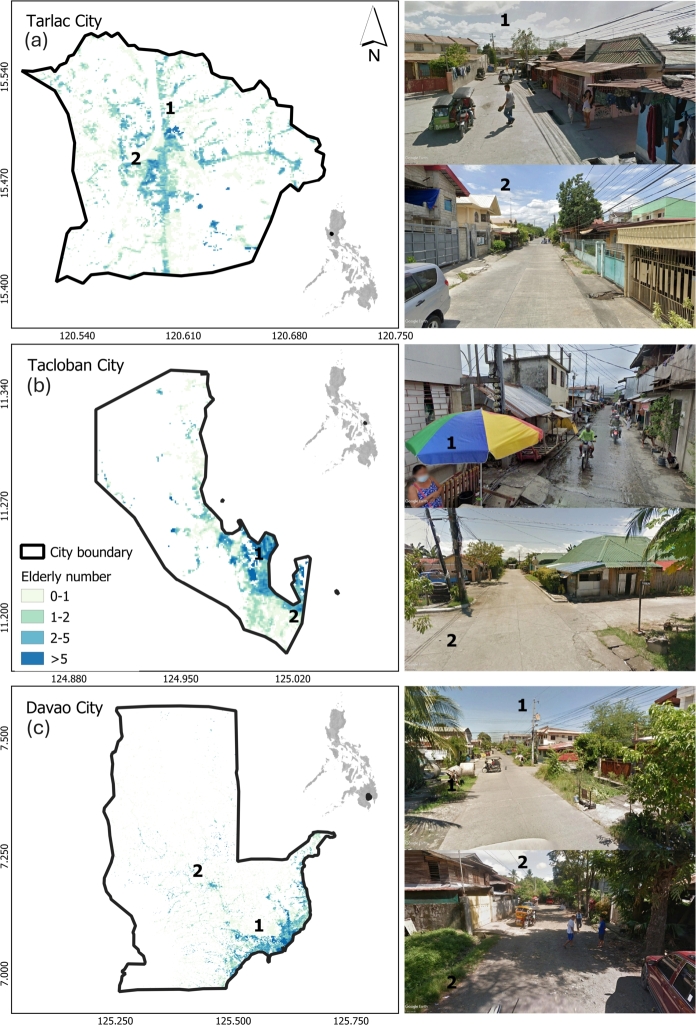
Geographic locations of the three study areas in the Philippines: (a) Tarlac, (b) Tacloban, and (c) Davao the potential number of older adults for every household. The older adults spatial data came from the Data For Good initiative [23]. This includes actual photos from Google Earth Street View showing the landscape and built environment of areas with high older adults. Note that most older adults reside within the city proper.

One city from each of the three major island groups in the Philippines was selected as the study area, namely (a) Tarlac City for Luzon, (b) Tacloban City for Visayas, and (c) Davao City for Mindanao (Fig. 1). Herein we respectively refer to them as Tarlac, Tacloban, and Davao. Tarlac is a landlocked area situated in Central Luzon and is 107 kilometers north of the Philippine capital, Metro Manila. The city comprises 76 smaller administrative units with arable lands suitable for producing vegetables and crops, especially rice and sugarcane. Meanwhile, Tacloban is a highly urbanized coastal city in the Eastern Visayas (Region VIII). The regional area is an island mainly surrounded by the sea, and it serves as the regional center. Lastly, Davao is a highly urbanized coastal city in Davao Region (Region XI) in Mindanao. It is the regional center for Mindanao and is composed of 182 *barangays*. Unlike the Eastern Visayas region, the Davao region is also located near a mountainous area with forest cover and land suitable for growing vegetable crops.

### 2.2 Body mass index classification

The household survey used was obtained from the study of the Department of Science and Technology - Food and Nutrition Research Institute (DOST-FNRI) in 2021-2022 as part of the project “Relationship of Body Composition to the Functional Capacity and Quality of Life of Older Filipinos in Selected Provinces in the Philippines”. The survey data was designed with the help of the project statistician and followed the World Health Organization classification (WHO) for categorizing the body mass index (BMI): underweight (<18.5 kg/m^2^), normal (18.5-24.9 kg/m^2^), and overweight and obese (overweight-obese) (>25.0 kg/m^2^) [24]. We used the WHO classification since it is the most widely used classification around the world to categorize BMI and in the DOST-FNRI National Nutrition Survey results. This can ease the comparison of results within the country survey results as well as with other countries by having used the same classification method.

### 2.3 Household surveys as presence-only and presence-absence data

**Figure 2:**
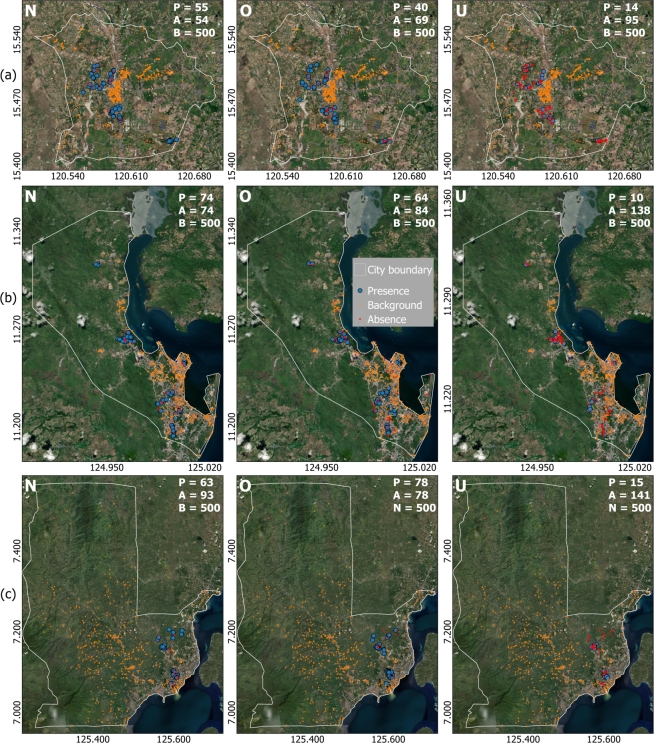
Locations and tally of older adults BMI class per city: (a) Tarlac, (b) Tacloban, and (c) Davao. *Barangay* or city sub-unit boundaries are also included in the image, together with satellite imagery to emphasize rural-urban landscapes and dominant land covers. Note that the older adults are only sampled in residential areas in Fig. 1.

Household surveys were used as both presence-only for MaxEnt models and presence-absence data for ML models. The survey followed a stratified random sampling of selecting the smaller city units locally known as *barangay*. Each *barangay* was assigned to one of four possible strata according to their dominant land cover, number of amenities, distance to the city center, number of older adults, and socio-economic status. For each stratum, the *barangay* of interest was selected randomly for the actual survey. The survey took place after pre-identifying households of older adults with local informants because of COVID restrictions. To minimize the potential effect of this convenient survey, we selected the MaxEnt background data with the same spatial distribution as the presence data [18]. We identified areas inside the older adults mask (“target group”) in Fig. 1 that are likely to have older adults=1. This ensures we are not assigning background data to households without or with >1 older adult, which may lead into having duplicated sample points. We also limit the background data in under-sampled areas determined as areas with high dissimilarity between survey points and environmental variables in feature space [25]. We used a threshold-based dissimilarity as a basis to delineate under-sampled areas using the approach of Meyer and Pebesma in 2021 [26] (Fig. S1). Fig. 2 shows the presence data and background locations of older adults across the three study cities in the Philippines: (a) Tarlac, (b) Tacloban, and (c) Davao. Each map shows the sampled locations of older adults in the *barangays* categorized by BMI class. The absence data (ML models) are data from the other two classes, e.g., presence=overweight-obese data and absence=underweight and normal data.

### 2.4 Environmental variables

The selection of environmental variables was primarily based on whether (1) they influence older adults' nutrition according to literature, (2) the availability of a published dataset, and (3) preferably high-resolution (30-100 m) data. A total of 43 variables were used for this study, all coming from open-source data that are produced either using remote sensing or OpenStreetMap data. Each variable is labeled uniquely in Table 1 and can be generalized into 11 categories: Nightlights, House to City, House to Roads, House to Amenities, Road Density, Greenness, Population, Building density, and Topography. Description about each variable is shown in the Table 1.

**Table 1:** List and data details of environmental variables used for predicting older adults BMI classes.

Main Variable	Unit	Sub-category	Source	Total Variables	Pixel size (m)	Reference
Night lights V1	Emission sources at night are detected by visible and near-infrared (VNIR)	Nightlights	DMSP-OLS	1	1000	Hsu et al. (2015) [29]
Night lights V2	Monthly average radiance composite images using nighttime data	Nightlights	NOOA-VIIRS	1	500	Mills et al. (2013) [30]
Proximity to main city V1	Distance (m) to the city centre	House to City	OSM	1	100	Haklay and Weber (2008) [31]
Proximity to main city V2	Distance to the main city every 1 km	House to City	OSM	1	100	Haklay and Weber (2008) [31]
Proximity to roads	Distance (m) to the nearest road	House to Roads	WorldPop	1	100	Tatem (2017) [27]
Proximity to highways	Distance (m) to the nearest main road (highway)	House to Roads	OSM	1	100	Haklay and Weber (2008) [31]
Proximity to food amenities	Distance (m) to food amenities location	House to Amenities	OSM	1	100	Haklay and Weber (2008) [31]
Road density (RD)	Length of roads divided by neighborhood area	House to Roads	OSM	1	100-1000	Haklay and Weber (2008) [31]
Normalized Difference Vegetation Index (NDVI)	Greenness indicator (0-1)	Greenness	GFC	1	30	Hansen et al. (2013) [32]
NDVI Texture	NDVI texture within a neighborhood	Greenness	GFC	5	30	Hansen et al. (2013) [32]
Built-up Index (BUI)	Built-up area indicator (0-1)	Greenness	GFC	1	30	Hansen et al. (2013) [32]
older adults population V1	Estimated number of older adults residing in each grid cell	Population	Meta	1	30	Meta (2022) [23]
older adults population V2	Estimated number of older adults residing in each grid cell	Population	WorldPop	1	100	Tatem (2017) [27]
Population demography	Changes in the estimated number of people residing in each grid cell in the past decades	Population	WorldPop	1	100	Tatem (2017) [27]
Population count 2015	Estimated number of people residing in each grid cell	Population	JRC	1	250	JRC (2015) [33]
Population count 2020	Estimated number of people residing in each grid cell constrained to buildings, adjusted using UN estimates and unconstrained	Population	WorldPop	3	100	Tatem (2017) [27]
Population count 2020	Estimated number of people residing in each grid cell	Population	GPW	1	1000	Centre and for International Earth Science Information Network (2018) [34]
Population density 2020	Total people over 100 ha	Population	WorldPop	1	1000	Tatem (2017) [27]
Residential building density (RD)	Number of residential buildings divided by neighborhood area	Population	OSM	4	100-1000	Haklay and Weber (2008) [31]
Residential area (RA)	Hectares of residential buildings in a neighborhood	Population	OSM	4	100-1000	Haklay and Weber (2008) [31]
Food amenities density (FD)	Number of food amenities divided by neighborhood area	House to Amenities	OSM	4	100-1000	Haklay and Weber (2008) [31]
Building density (BD)	Number of buildings divided by neighborhood area	Building density	OSM	4	100-1000	Haklay and Weber (2008) [31]
Building area (BA)	Hectares of buildings in a neighborhood	Building density	OSM	4	100-1000	Haklay and Weber (2008) [31]
Elevation	Meters Above Sea Level	Topography	SRTM	1	30	Van Zyl (2001) [35]
Slope	Degrees	Topography	SRTM	1	30	Van Zyl (2001) [35]

The raster data of all environmental variables were resampled to 100 m and masked using areas with potential older adults. The latter is defined as areas both identified as built-up and areas with at least one living older adult, both considering two gridded demographic datasets [23], [27]. All raster pre-processing was accomplished using *R* statistical software *terra* package [28].

### 2.5 Spatial machine learning (ML) models

We implemented an ML ensemble approach on top of individual ML models (base learners) under the context of model generalization [36]. The motivation for this approach lies in the proven ability of ensemble ML methods to outperform individual models. We used Random Forest (RF), Artificial Neural Networks, a Gradient Boosting model, and two MaxEnt models as base learners. Commonly employed in the literature, these models contribute diverse strengths in capturing complex patterns and handling non-linear relationships [37]; further details are provided in the Supplementary Materials. The predictions of each base learner become variables to a final model called a meta learner. Here, we chose RF as the meta-learner, given some correlation (0.4-0.7 R^2^) among individual ML predictions from the initial analysis. Random Forest (RF) reduces overfitting by generating non-correlated decision trees through data bootstrapping and feature randomness, allowing the ensemble to smooth out individual tree errors and improve generalization. The ensemble includes the final prediction of each BMI class probability and associated standard deviation layers, originating from individual ML models' uncertainty. We assigned malnutrition hotspots as areas above 0.8 probability and mapped them separately for underweight and overweight-obese. Finally, the hotspot areas were summarized and their area totals per city sub-units or *barangays* were reported. All ML workflow was implemented using the *SDMTune R* package for computational efficiency compared to similar implementations [38].

### 2.6 Model evaluations

The validity of the models was evaluated using random five-fold cross-validation, particularly the Area Under Receiver Operating Characteristic (AUC) [39] and its mean after cross-validation. The AUC metric measures the separability between two probability (Pr) classes, i.e., true positives (TP) and false positives (FP). An AUC of 0.7-0.9 indicates moderate model performance, while >0.9 indicates high performance. See equation (1), where Pr[TP] is a function of v=Pr[FP]. The evaluation is complemented by the variable importance results (explained next sub-section) that can identify which base learner influences the ensemble model the most.

(1) $$\mathrm{AUC}=\int_{0}^{1}\mathrm{Pr}[\mathrm{TP}](v)dv$$

True Skill Statistics (TSS) is another commonly used performance metric for binary classification models. Like AUC, TSS also measures the separability between two probability classes (TP and FP). However, TSS also takes into account the true negatives (TN) and false negatives (FN) and is calculated in equation (2) below:

(2) TSS=TP/(TP+FN)−FP/(FP+TN)

The TSS ranges between -1 and 1, and a TSS of 0.4–0.6 indicates moderate model performance, while >0.6 indicates high performance.

### 2.7 Variable decorrelation and model tuning

While several ML models such as MaxEnt and RF, are less prone to variable multicollinearity compared to other machine learning models, we were cautious of this notion. Hence, we performed a cross-correlation analysis among the variables. After learning that many variables are correlated and as advised in Phillips et al. 2017 [40], we decided to apply a variable decorrelation step. Among possible variable combinations, the step correlates two variables. For those pairs with >0.8 correlation, we selected the variable that is more sensitive to the model, i.e., model AUC decreases if the selected variable is removed. On average, 22 out of 43 variables remained after the decorrelation step.

Another intermediate step was tuning the hyperparameters of every ML model. We applied a grid search approach of selecting the hyperparameter combination that yielded the highest AUC among potential combinations. What defines the combination are user-defined parameter values aside from the default ones. We used our judgment to set the preliminary parameters.

### 2.8 Variables affecting malnutrition

The impact of each variable on the ML models was assessed using the results of individual ML models in two ways. This step reveals sensitivity of each variable to malnutrition, including potential effects of uncertainties inherent to the variable dataset. First, jack-knife tests were implemented where models are fitted using individual variables. The resulting AUC for each model indicates the variable importance. Second, data permutation tests were done where values of a variable were shuffled and fed to another model to be run. The AUC before and after the permutation was then recorded and each variable's relative contributions (0-100%) were computed. The permutations were iterated ten times and the ten relative contributions were averaged. We summarized the jack-knife and permutation results for each generic variable category (see Table 1) and compared the results among cities. This needed normalization of the data permutation results. Comparisons among results per city would indicate the consistency in spatial patterns of malnutrition. The consistency was measured by the average absolute Pearson correlation coefficient (*r*) of the correlation results among three city pairs. Such metric measures the strength and direction of the linear relationship between two continuous variables. Lastly, we plotted the variable response curve, which measures how changes in a certain variable affect malnutrition while holding all other variables in the model at their actual values. This can be useful for understanding the overall effect of the independent variables on malnutrition.

### 2.9 Malnutrition spatial distributions and hotspots

The last steps were to map the probability of normal, overweight-obese, and underweight older adults using the ensemble models. These maps were used to delineate malnutrition hotspots spatially based on a probability of occurrence threshold >0.8 in combination with areas supported by the sample defined in Fig. S1. This step produced hotspots based on thresholding alone (Hotspot 2) and the combined thresholding and sampling considerations (Hotspot 1). Here, we focused on overweight-obese older adults given the policy concerns to address obesity and since our preliminary results revealed a low probability of underweight older adults in the three cities. The hotspot maps are also termed as realized distribution maps in the context of species distribution modeling. Map labels “Overweight” correspond to “Overweight-obese”. Lastly, we also produced associated standard deviation layers based on all possible probability distribution maps of each model.

## 3 Results

### 3.1 Spatial model evaluation

The evaluation of each ML model measured by the average AUC and TSS values from every held-out data of the cross-validation is shown in Table 2. Overall, the models perform decently with AUC values ranging from 0.76 to 0.93 and TSS from 0.45 to 0.81. These results are within the accepted thresholds that indicate that the model is performing better than random chance, i.e., >0.7 (AUC) and >0.5 (TSS). Per city results, the Tarlac models are the best-performing models, having AUC=0.92 and TSS=0.75, on average. Tarlac is followed by Davao (AUC=0.85 and TSS=0.62) and Tacloban (AUC=0.78 and TSS=0.48). Regarding the results per BMI class, the models for underweight obtain the highest evaluation metrics, especially for Tarlac and Davao. The results of models for normal and overweight-obese older adults are almost the same.

**Table 2:** Average AUC and TSS from the cross-validation results of the ensemble model per BMI class and city.

City and BMI classification	AUC	TSS
***Tarlac***	***0.92***	***0.75***
Normal	0.91	0.69
Overweight-obese	0.93	0.74
Underweight	0.93	0.81
***Tacloban***	***0.78***	***0.48***
Normal	0.77	0.45
Overweight-obese	0.76	0.45
Underweight	0.79	0.54
***Davao***	***0.85***	***0.62***
Normal	0.84	0.59
Overweight-obese	0.81	0.54
Underweight	0.9	0.72

The cross-validated results (ensemble model) have 0.03 to 0.30 higher AUC than the results when using base learners. The same ensemble model is also higher than a model without hyperparameter tuning and decorrelation (Section 2.3) but only by 0.03 AUC. The Table 2 results of AUC and TSS are linearly related (r=0.97). Results of individual models are shown in Fig. S4 wherein the ensemble models show higher AUC than all base learners.

### 3.2 Environmental drivers of normal, overweight-obese and underweight older adults

**Figure 3:**
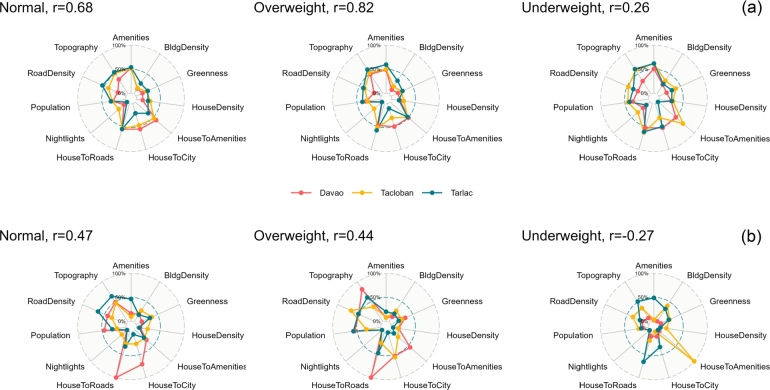
The AUC values (multiplied by 100) after the jack-knife tests where models are run with individual variables (a) and the relative variable importance after data permutation (b). Pearson's correlations (r) are also shown as the average of the three correlations among variables for every city. Note that variable importance is aggregated for each sub-category in Table 1 for better results interpretation and visualization.

The results of the jack-knifing and data permutation tests to evaluate variable importance are shown in Fig. 3. Both importance metrics show similar important variables except for the data permutation results for underweight. The house-to-roads and house-to-main city distances appear to be the most important variables for most models. The next important variables are topography, road density, and the number of amenities. Of least importance are night lights and greenness. These observations are more pronounced in the results of the jack-knifing test (Fig. 3) than in the data permutation (Fig. 3b). The common, most important variables among the three cities are also evident, especially for models of normal and overweight-obese. The jack-knife test shows more consistency among cities as indicated by Pearson's correlation average of the possible three correlations among cities (overweight-obese=0.82 and normal=0.68, Fig. 3a). The consistency between important variables among cities is almost similar between normal and overweight-obese. The important variables of the model for underweight older adults among cities are highly inconsistent when using the permutation test (Fig. 3b).

**Figure 4:**
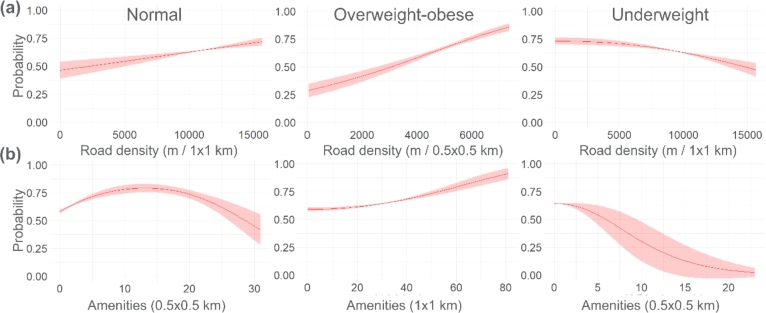
Response curves of the variable house to roads (km) from normal, overweight, and underweight models in Tarlac (a) and a number of amenities over different buffer distances in Tacloban (b). The filled areas parallel to the line graph are ranges due to cross-validation. The graphs below concern the number of amenities in the neighborhood.

However, the consistency among important variables of normal, overweight-obese and underweight models does not mean that the variables have the same association with malnutrition as indicated by the variable response curves (see example variable, Fig. 4). The figure shows that being overweight-obese is proportionally related to road density (Fig. 4a). This means that neighborhoods with the densest roads most probably have overweight-obese older adults. This observation is less pronounced for normal older adults and the opposite for underweight older adults. In terms of the number of amenities in Fig. 4b, areas with a high number of amenities over 100 ha (e.g., 80) have a very high probability of having overweight-obese older adults. This contrasts with the probability of having normal and underweight older adults. The other response curves can be seen in Fig. 4b, where shorter house-to-road distances and flat and lowland topography are positively related to being overweight. The rest of the response curves are shown in Fig. S2.

### 3.3 Probability distribution maps and hotspots of malnutrition

**Figure 5:**
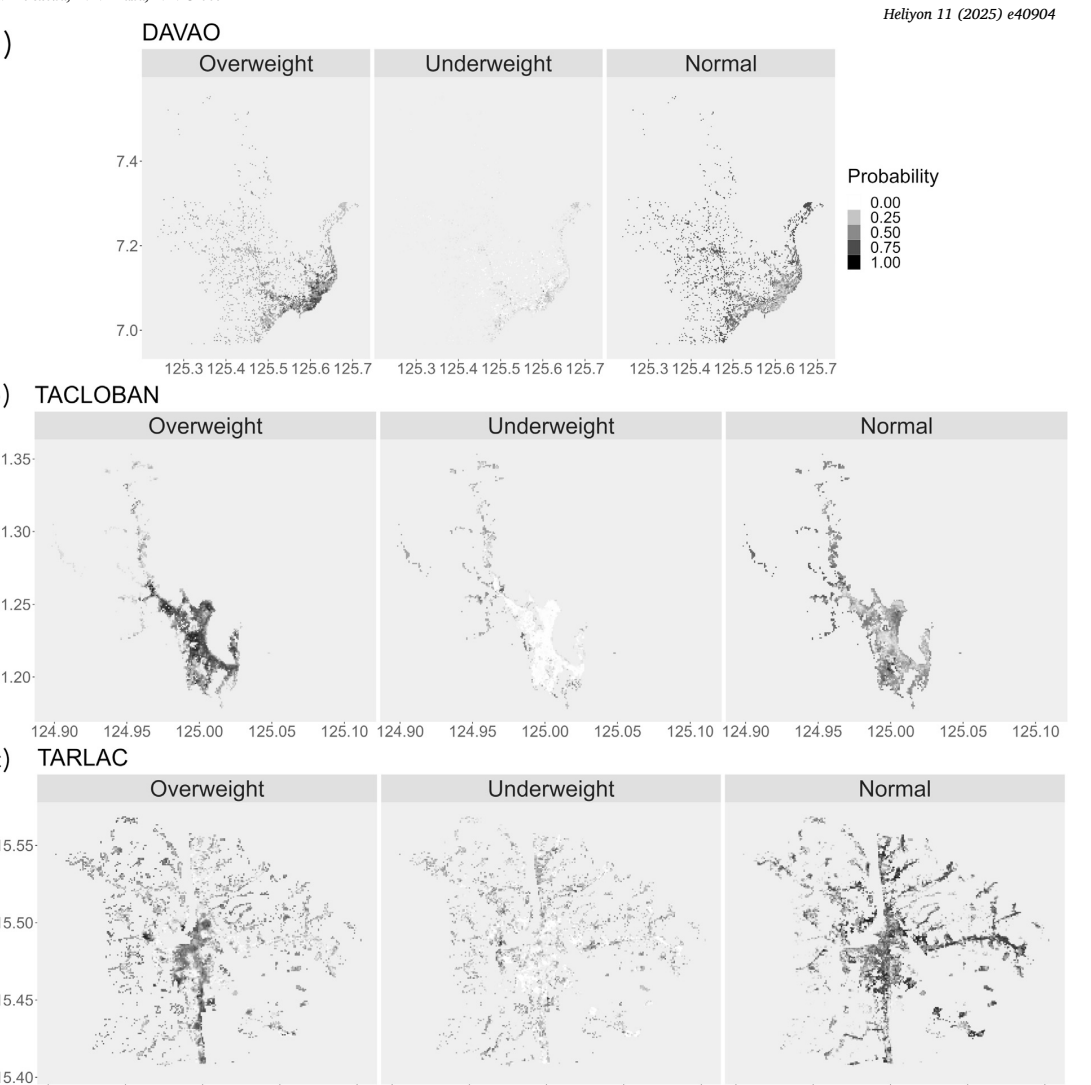
Presence probability map showing the distribution of older adults by BMI classification in (a) Davao, (b) Tacloban, (c) Tarlac. Overweight-obese older adults are mostly found in urban areas, especially city centers, while underweight individuals are rare and scattered. These patterns reveal clear differences between urban and rural environments in shaping older adults' BMI status.

The spatial predictions of normal, overweight-obese, and underweight older adults for the cities of (a) Davao, (b) Tacloban, and (c) Tarlac are shown in Fig. 5. The highest probability of occurrence is observed in maps showing overweight-obese older adults, while the lowest probability is observed in maps of underweight older adults. The predictions for normal older adults are mostly moderately probable. There is a strong spatial pattern between the probabilities of the three BMI classes. Most overweight-obese older adults are seen in the main city, while older adults with normal BMI seem to be evenly spread across both urban and rural areas. This is the opposite of the underweight older adults, who are very scarce. These observations are consistent among cities. Most older adults are predicted to be overweight-obese in Tacloban, normal in Tarlac, and a balance between overweight-obese and normal in Davao.

## 4 Discussion

### 4.1 Spatial model evaluations

The spatial models performed reasonably well (AUC=0.76-0.93 and TSS=0.45-0.81). The highest evaluation results were observed from models for underweight older adults. This happens despite the lowest sample size for the underweight and an imbalanced number of presence and absence samples. This can be an indication that the models for underweight overfit [17]. While ensemble modeling can help minimize model overfitting despite using small samples, other factors can influence models to overfit such as the high number of predictor variables, especially correlated ones [20]. This may be the case even though we already removed around 24 out of 47 possible variables due to multicollinearity. Hence, the obvious intervention to minimize the sampling issue is to increase the sample size. Here, we delineated areas where the focus of data collection should be using the area of applicability approach [26]. We also discarded these under-sampled areas when delineating the overweight-obese hotspots as shown in Fig. S1.

The literature is not in full consensus on whether ensemble modeling is the preferred model of choice in the context of species distributions where under-sampling is always an issue. Breiner et al. 2015 [20] demonstrated how ensemble modeling is useful even when using sample sizes ranging from 10-104. Valavi et al. 2022 [19] also showed the superiority of ensemble models over individual models such as Random Forest (RF), support vector machines, and generalized linear models, among others. The latter study also showed that tuned and modified (balanced presence-absence samples) models, particularly from RF, can have a similar model performance to ensemble models. Contrary, recent studies stated that when fitting distribution models with small samples, which can also be the case for underweight older adults, ensemble modeling can be outperformed by MaxEnt [41] and boosted regression trees (BRT) [42]. Here, we used MaxEnt and MaxNet (presence only) and RF, BRT, and neural networks (presence-absence) and found the superiority of ensemble models when comparing cross-validation results among the individual models [20]. The strengths of both presence-only and presence-absence models can also be captured in terms of increasing the reliability of predicting occurrences while having actual data to predict absences [43]. Moreover, we applied a generalization framework as an ensemble method, unlike the usual ensembling methods that rely on averaging and weighted averaging based on AUC values. Here, model stacking was used (Section 2.2) and an RF meta-model was used to avoid multicollinearity issues among predictions of individual models (base learners) [44].

We applied 5-fold random cross-validation to assess model accuracy, as it is particularly recommended for small sample sizes. The choice of cross-validation method can also depend on the sampling design [45]. While we applied stratification and followed literature recommendations to limit the background data area in our presence-only models (using an older adults mask), COVID-19 restrictions resulted in purposive sampling. Nevertheless, it is essential to account for this and apply a more appropriate cross-validation approach, especially when incorporating additional samples from secondary sources not included in the original sampling design.

### 4.2 Environmental variables that drive malnutrition

The house-to-roads and house-to-main city distances appear to be the most important variables for most spatial models (Fig. 3) and they are inversely related to the older adults being overweight-obese (Fig. 4). Neighborhoods with older adults that are relatively far from roads have less access to the main city in general. Farther distances to the cities were found to be a limiting factor for overweight-obese cases [11], [16]. In developing countries such as the Philippines, this is mostly because physical activity is linked to work, wherein there is higher energy expenditure in the workplace in rural areas than in urban places [46]. Additionally, main cities harbor the cheapest and most unhealthy foods in developing countries [47], [48]. We further found that many food amenities (mostly fast-food establishments) were driving the likelihood of older adults being overweight-obese. Also located in cities are the densest roads which we found to drive being overweight-obese. These findings provide additional evidence that environmental factors driving malnutrition coexist.

While many studies like Sun et al. 2020 [16] mentioned road density being a limiting factor of obesity because of its association with physical activity (walking), this is likely not the case in tropical developing countries like the Philippines. The country's common city public transport vehicles (*jeepneys* and *tricycles*) are very active and people prefer to access them instead of walking. This is because most city streets are not conducive for side walking due to poor infrastructure, crime incidents, air pollution and flood [49], [50]. In addition, the study of Mateo-Babiano and Ieda in 2007 [51] found that Asian pedestrian roads are usually a venue for commerce and exchange and a place to socialize, which is directly correlated between walking and nonmovement spaces. These areas attract high pedestrian volume and usually have a greater tendency for nonmovement activities. We also think that the COVID-19 restrictions resulted in inactive physical activity of older adults in general. We found no association between neighborhood greenness and older adults' malnutrition. This can be a result of using high-resolution inputs (1 ha) where mostly built-up signals are captured.

The jack-knifing and data permutation tests to evaluate variable importance (Fig. 4) showed similar important variables except for the data permutation results for underweight. These two are both resampling methods, but the way they resample data is different (Section 2.3). Moreover, data permutation focuses on the importance of individual variables, whereas jack-knifing focuses on the overall performance and stability of the model. While most of the studies focused on using one variable importance method, several studies demonstrated how jack-knifing and data permutation results are complementary, e.g., De Cauwer et al. (2014) [52] and Stoetzel et al. (2020) [53]. These interpretation tools, along with the response curves, are helpful in interpreting machine learning models.

### 4.3 Spatial patterns of malnutrition among cities

The spatial patterns of overweight-obese older adults (within and nearby cities) suggest that urban areas are obesogenic. This supports the research findings wherein the prevalence of being overweight and obese is higher in urban areas. The data show that older adults with normal BMI seemed to be everywhere, while underweight older adults were scarce. These observations mirror national results where the underweight is the lesser and the overweight and obese are getting higher [54], [55]. Per city, the older adults in Tacloban have the highest prevalence of overweight-obese, normal in Tarlac, and a balance between overweight-obese and normal in Davao. Such inter-city results can be related to the main economic activity of the cities, wherein Tarlac is mostly an agricultural city. At the same time, Tacloban and Davao are coastal cities - the latter has more dynamic economic activity in the main city, but 90% of the area is rural.

### 4.4 Study novelty and limitations

Here we demonstrated the first neighborhood level (1 ha) mapping in tropical developing countries and most likely globally. This is made possible by remote sensing data, all open-source and mostly high resolution and the updated OpenStreetMap database where we obtained housing and food amenities locations. This means the methodology can be upscaled not only within the Philippines but also in other countries. Previous mapping efforts in the Philippines provided maps at coarser spatial resolutions (e.g., Dahly et al. 2013 [7] and Salvacion 2017 [8]. Having a spatially detailed map, especially for overweight-obese and underweight older adults, is key to more focused interventions and awareness of priority areas. The latter can be provided by the hotspot maps (realized distribution) in Fig. S3. The maps need to be communicated to the city and local governments to have a social impact on Urban Planning. Local governments could use this data to create specific programs or facilities that would encourage older persons to engage in physical activity, as well as a model for targeting nutrition and emergency programs.

We showed how normal and overweight-obese older adults co-exist in neighborhoods and cities but found very few underweight older adults. The improbable occurrences of underweight in the three cities can be caused by unreliable model predictions due to small samples and model overfitting. The effect of this issue is depicted in Fig. S1 (red areas). It can also be possible that underweight older adults (BMI <18.5 kg/m^2^) rarely exist, especially in areas that are supported by the sample. Given the changing environment and land uses, these city findings can be a baseline for future studies on how older adults' nutrition thrives.

This study is cross-sectional (static), meaning it cannot infer causal relationships between older adults' malnutrition and the environment and cannot be used to study malnutrition trends. Regarding the latter, there is a plan to include more cities in the nationwide nutritional surveys and we also identified priority areas for additional samples in the three case cities. Once there are more samples in different years, we can explore developing spatio-temporal models that can predict the occurrence of normal, overweight-obese, and underweight older adults over time. Moreover, we can develop generalized models (one model) that can be used to predict malnutrition in more cities. A representativeness analysis of the older adults' survey locations is needed to make sure that target cities have the same environmental features as the cities with sample data to avoid extrapolation (unreliable predictions).

Another limitation was the multiple factors that influence the relationship between BMI and the older adult population, including changes in population demographics, lifestyle behaviors, healthcare advancements, and other societal factors. Thus, the association of one clear BMI cut-off is probably not stable within populations over time. In the same way that there are environmentally determined differences in these associations across different population groups, these associations also vary within populations according to environmental changes and nutritional transitions [56].

We used WHO classification in categorizing the BMI of older adults, even though Filipinos and most Asians have a higher percentage of body fat than other ethnic groups. We relied on a generalized classification for cross-population comparisons.

## 5 Conclusions

This study aimed to associate environmental variables affecting the body mass index of older adults while mapping the the occurrence probability of normal, overweight-obese and underweight older adults at neighborhood levels (1 ha). The methodology is data-driven (open access remote sensing and OpenStreetMap data) and based on an ensemble machine learning of different presence-only and presence-absence spatial models. We conclude that:

1. Spatial models tend to overfit despite being subjected to good practices when producing background data, model tuning, variable decorrelation, and appropriate cross-validation. This was shown by the results of models used to predict underweight older adults with the highest evaluation results despite having the lowest and unrepresentative samples. The only option to mitigate this issue is to add more samples, preferably to the priority areas in Fig. S1.
2. The two methods (jack-knifing and data permutation) of quantifying the importance of environmental variables were mostly complementary and both revealed consistent important variables among cities and BMI classes. However, in order to interpret and understand the malnutrition-environment associations, response curves were needed.
3. Short distances to the roads and main city were positively associated with overweight-obese older adults, the latter being a common finding in the literature. Road density was found not to be a limiting factor of overweight-obese older adults, contrary to the findings in developed Western countries. Factors such as active public transport, weather, crime, walkability, and even COVID-19 restrictions during the time of the surveys all influence the result.
4. The spatial model of the older adults in the three Philippine case cities confirmed the increasing obesity prevalence in the country especially in urban areas. Here, we recommend that city and local governments to closely look at the walkability of the built environment in their areas.

## Ethics statement

The research study received ethical clearance (FIERC-2021-017) from the FNRI Institutional Ethical Review Committee. Informed consent was obtained from all study participants.

## CRediT authorship contribution statement

**D.K. Mendoza:** Writing – review & editing, Writing – original draft, Validation, Methodology, Conceptualization. **A.B. Araza:** Writing – review & editing, Writing – original draft, Methodology, Investigation, Conceptualization. **L.D. Groot:** Writing – review & editing. **M. Mensink:** Writing – review & editing. **R.C. Tan:** Writing – review & editing, Investigation.

## Declaration of Competing Interest

All authors declare that they have no known competing financial interests or personal relationships that could have appeared to influence the work reported in this paper. R.C. Tan reports financial support for the research was provided by the Republic of the Philippines Department of Science and Technology Grants in Aid. The funder had no role in study design, data collection and analysis, decision to publish, or preparation of the manuscript.

## Data Availability

Data and codes will be made available upon reasonable request.

## Appendix A Supplementary material

MMC The supplementary materials provide description of spatial variables used; information about the base learners used; and results and analysis related to the areas supported by the household samples, response curves for all study sites, hotspot maps of overweight-obese older persons and model results for all base learners. MMC
