# Supplementary material for: Environmental factors affecting the BMI of older adults in the Philippines spatially assessed using machine learning
Source: Heliyon. 2024 Dec 6;11(1):e40904. doi: 10.1016/j.heliyon.2024.e40904 (PMC11719313; doi:10.1016/j.heliyon.2024.e40904)
Supplement: MMC — The supplementary materials provide description of spatial variables used; information about the base learners used; and results and analysis related to the areas supported by the household samples, response curves for all study sites, hotspot maps of overweight-obese older persons and model results for all base learners. [file mmc1.pdf]

Supplementary materials: Environmental factors affecting the BMI of older adults in the Philippines spatially assessed using machine learning

Description of spatial variables used

One of the main datasets used was the WorldPop dataset, a collection of high-resolution gridded demographic data [5]. We used three versions of their population data year 2020, including one where each population estimate is rectified with actual settlement buildings. We also used two other gridded population datasets from different sources. Another primary data used was the OpenStreetMap (OSM) crowd-sourced dataset [1]. The OSM dataset enabled us to derive area and density maps of buildings, settlements, amenities, and roads that indicate whether a neighborhood is urbanized. These area and density variables were iterated over neighborhood sizes of 1-10 ha. Similarly, the neighborhood greenness, indicated by the Normalized Vegetation Difference Index (NDVI) and Built-up Index (BUI), was considered in different neighborhood sizes by computing grid textures. The indices were derived using Landsat satellite and accessed from the Global Forest Change [2]. Their values over neighborhoods were estimated using Grey-Level Co-Occurrence Matrix [4]. Another set of variables concerns the accessibility of food and health amenities to older adults. We computed distances to the main city, food amenities, and roads. Afterward, we included topographic variables such as slope and elevation based on a Digital Elevation Model. The details of these main variables and other variables used are shown in Table 1.

Base learners

Maximum entropy

Models of MaxEnt and its generalized linear model implementation, MaxNet, were used. The former is modified as an Inhomogeneous Poisson process that predicts the probability of presence (instead of relative presence), hence the MaxNet implementation. At the same time, MaxNet extends this functionality to fit MaxEnt models using logistic regression. Herein, MaxEnt and MaxNet are called ME and MN, respectively. Their core algorithm is based on a Bayesian framework that generates predictions depending on how the presence-background data represent the environmental variables. The Bayesian function of MaxEnt allows prediction from non-sampled areas after

defining a probability distribution with maximum entropy ("MaxEnt distribution"), hence its potential geographic distribution. We implemented MaxEnt under the guidelines in [3], particularly in dealing with sampling bias (see Household Survey section) and correlated environmental variables (see Variables Decorrelation section).

Random Forest

Random forest (RF) model is a bagging model of decision trees where trees are grown simultaneously and uncorrelated. The first step of RF is bootstrapping the training data, i.e., a training fold that is 80% of the total data for five-fold cross-validation. Every bootstrap sample is the basis for growing trees, starting with the root node. Subsequent nodes are created using a subset of the bootstrap sample based on a limited set of variables; the latter step minimizes model overfitting. After every node splits, leading to the final node, the final predictions for each tree are tallied to form the final classification. We considered each tree vote instead of the final vote to derive a class probability.

Gradient Boosted Model

Gradient boosting model is also based on decision trees, but trees are boosted or grown sequentially instead of bagging. Tree boosting allows the model to improve after every result from the previous model iteration and their residuals. The residuals are used as weights to allow any weak classifier to auto-improve. This error minimization step, also known as optimization, is based on a threshold based on the "gradient descent" of a loss function such as Mean Square Error. Gradient boosting models also predict beyond the range of training data and also implement data imputation within the training data.

Artificial Neural Network

Artificial Neural Network belongs to a bigger set of ML models based on neural networks that mimic how the human brain functions (neurons) to learn from input data and its patterns. The components of an NN model consist of the input layer, hidden layers, and an output layer. The variables and hence the input layers are inter-connected to the first set of hidden layers with initial weights. The first hidden layer can be connected to a second set of hidden layers until the output layer or the target malnutrition class is reached. The connection from the input to the output layer is called a forward pass, which

results in a classification (i.e., binary in our case). Within these connections, the weights are adjusted to satisfy the thresholds in the output layer. This iterative step, called backpropagation, is also based on a loss function.

The syntax of the base learners using the SDMTune package can be found here `https://cran.r-project.org/web/packages/SDMtune/vignettes`

Figure S1. Areas supported by the household survey (background data of presence-only data excluded) sample based on feature space dissimilarity index. Red pixels depict areas that need more samples.

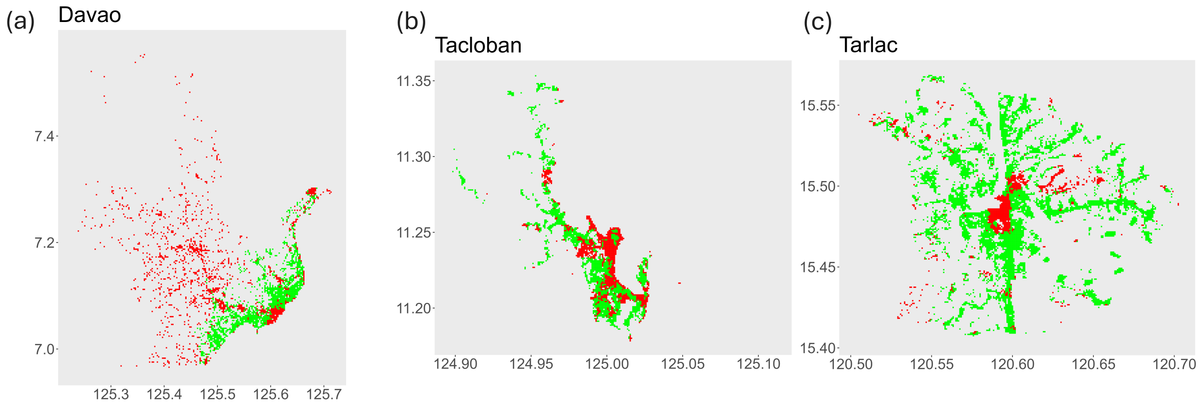

Figure S2. Response curves of the variable for the study areas using MaxEnt models

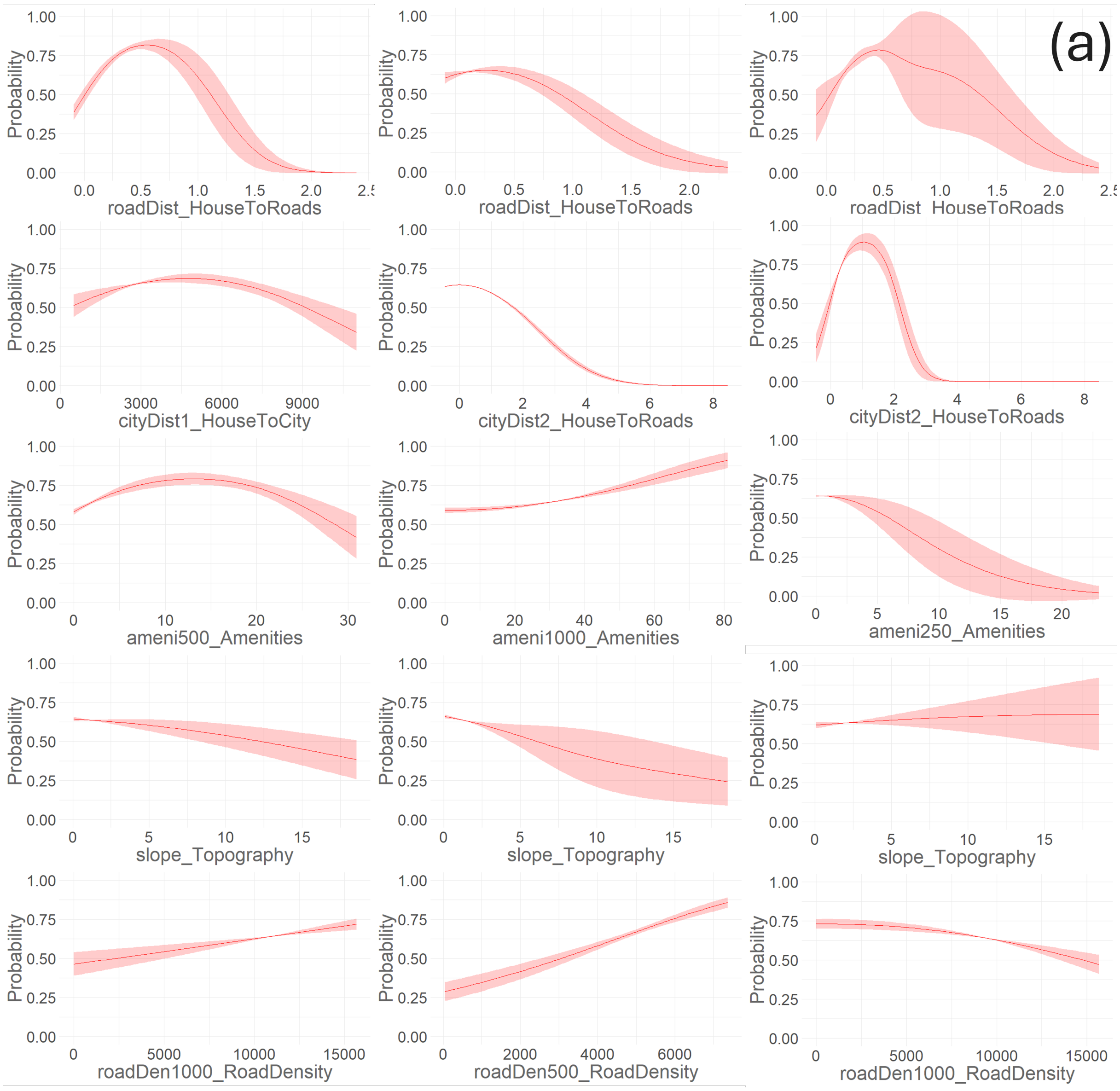

Figure S2(a). Response curves of the variable for Davao using MaxEnt models

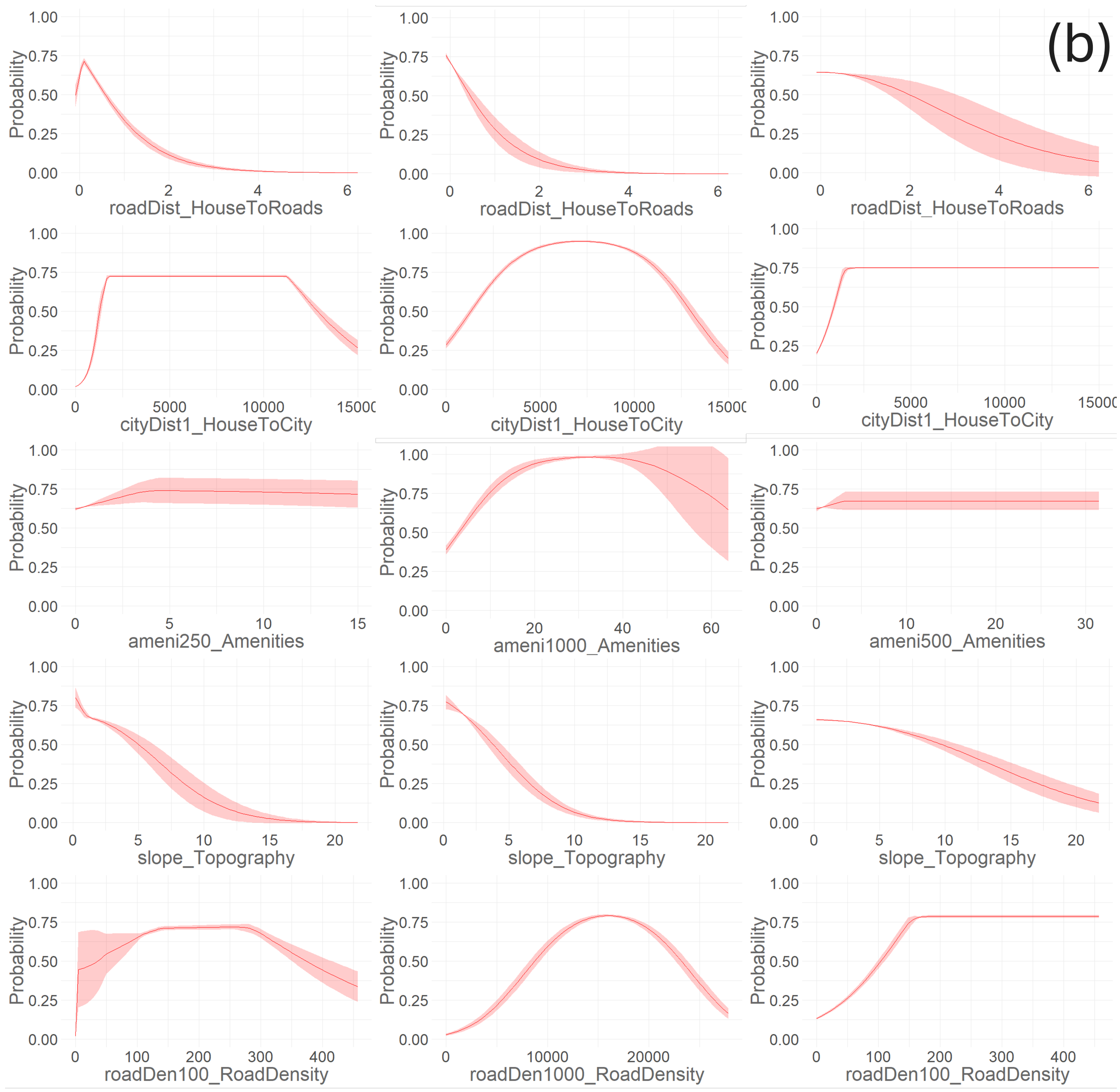

Figure S2(b). Response curves of the variable for Tacloban using MaxEnt models.

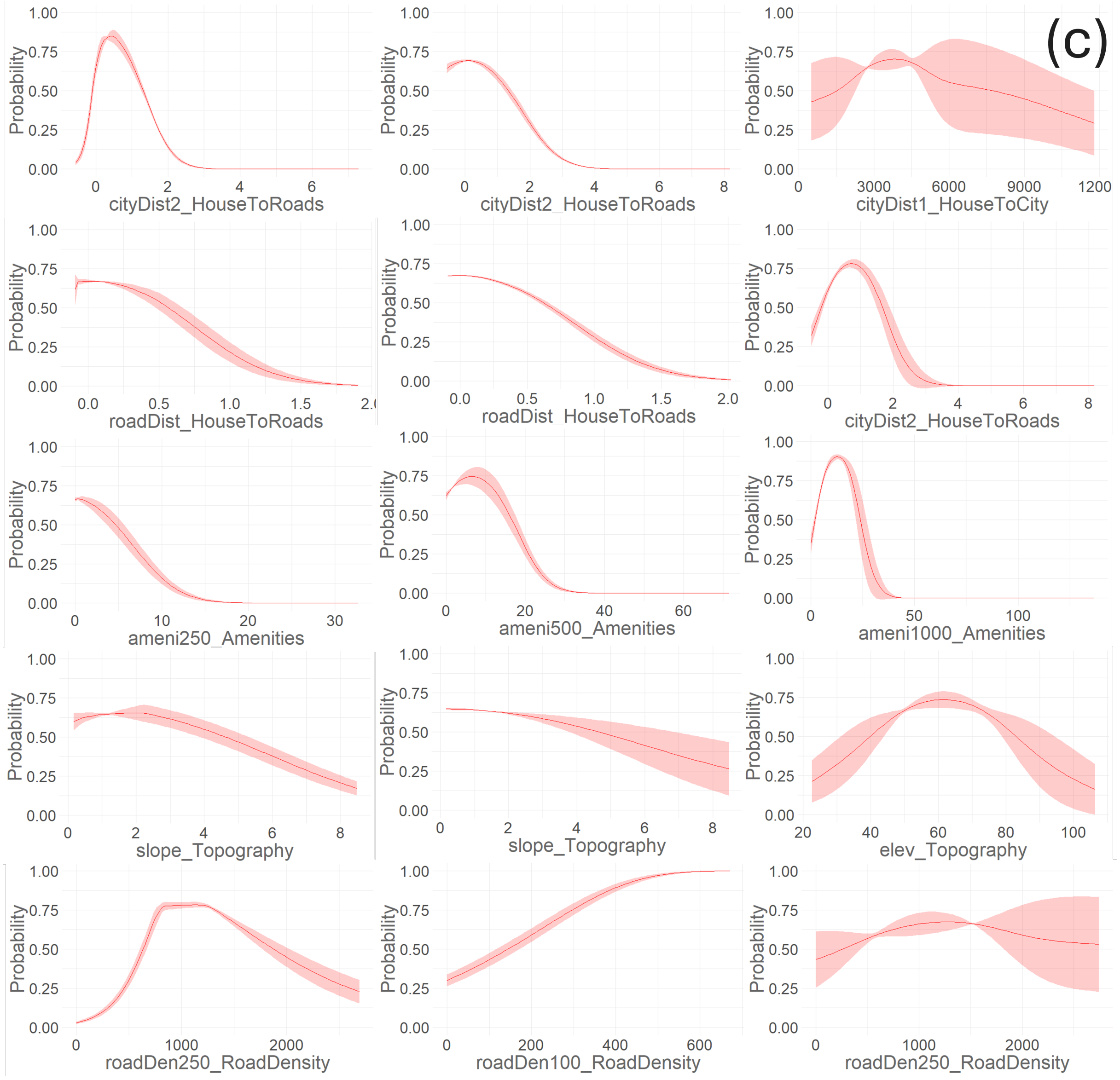

Figure S2(c). Response curves of the variable for Tarlac using MaxEnt models.

Figure S3. Hotspot maps of overweight-obese older adults for Davao (a), Tacloban (b), and Tarlac (c)

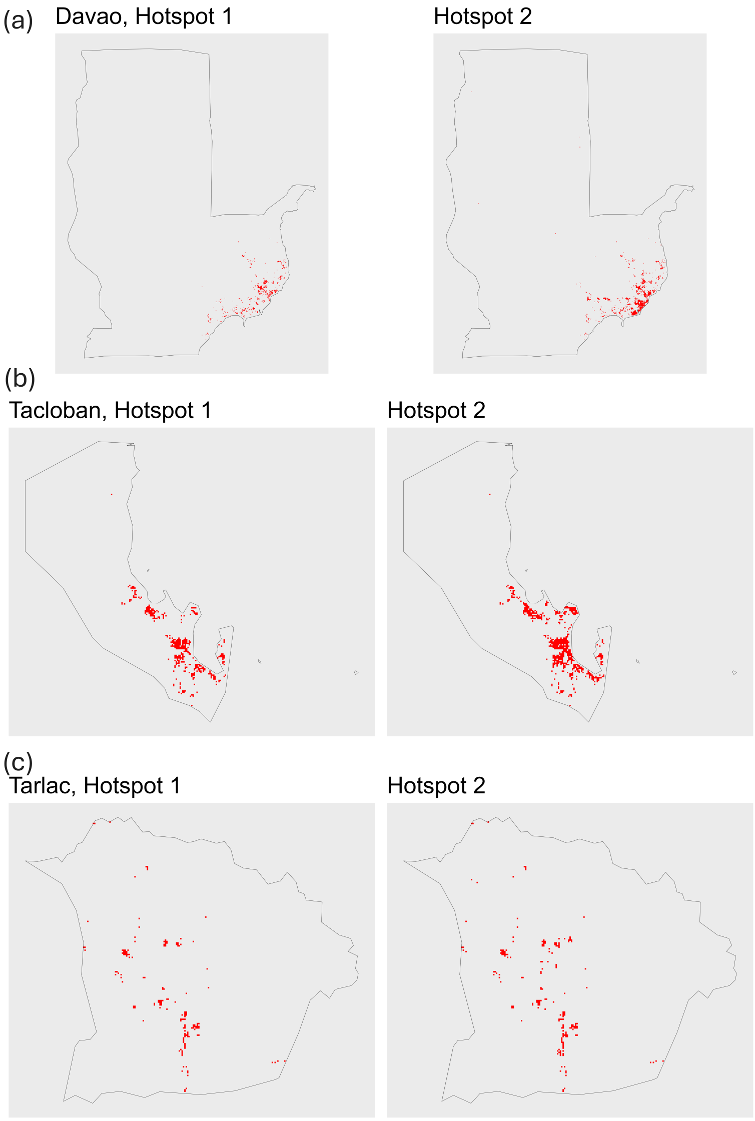

83 *Figure S4. AUC results of base learners and the meta learner.*

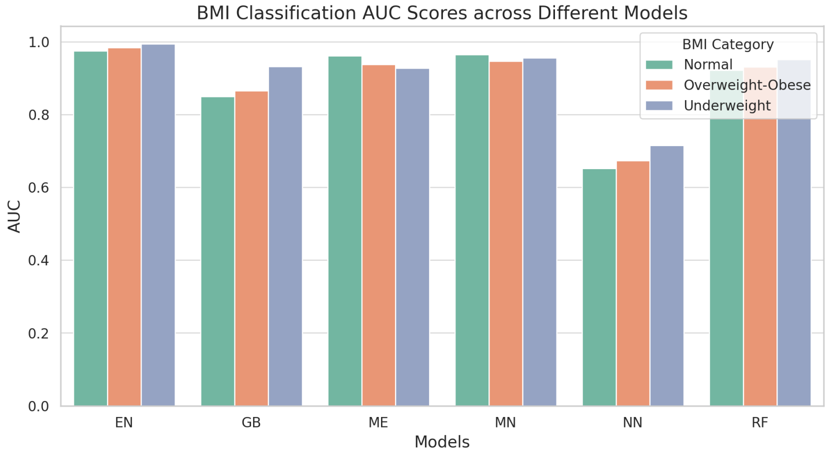

References

[1] Haklay, M. and Weber, P. (2008). Openstreetmap: User-generated street maps. *IEEE Pervasive computing*, 7(4):12–18.

[2] Hansen, M. C., Potapov, P. V., Moore, R., Hancher, M., Turubanova, S. A., Tyukavina, A., Thau, D., Stehman, S. V., Goetz, S. J., Loveland, T. R., et al. (2013). High-resolution global maps of 21st-century forest cover change. *science*, 342(6160):850–853.

[3] Merow, C., Smith, M. J., and Silander Jr, J. A. (2013). A practical guide to maxent for modeling species' distributions: what it does, and why inputs and settings matter. *Ecography*, 36(10):1058–1069.

[4] Mohanaiah, P., Sathyanarayana, P., and GuruKumar, L. (2013). Image texture feature extraction using glcm approach. *International journal of scientific and research publications*, 3(5):1–5.

[5] Tatem, A. J. (2017). Worldpop, open data for spatial demography. *Scientific data*, 4(1):1–4.
